# Effect of replacing cottonseed meal with canola meal on growth performance, blood metabolites, thyroid function, and ruminal parameters of growing lambs

**DOI:** 10.1007/s11250-023-03528-0

**Published:** 2023-03-18

**Authors:** Haitham M. M. Agwa, Hisham M. Saleh, Mohamed Salah Ayyat, Gamal A. Abdel-Rahman

**Affiliations:** 1 Biological Applications Department, Nuclear Research Center, Egyptian Atomic Energy Authority, Cairo, Egypt; 2 Department of Animal Production, Faculty of Agriculture, Zagazig University, Zagazig, Egypt

**Keywords:** Dietary protein, Sheep, Oilseed meals, Fermentation, Metabolism

## Abstract

The objective was to clarify the impact of replacing cottonseed meal with canola meal (CM) on growth performance, blood metabolites, thyroxin function, and ruminal parameters of growing lambs. Twenty-four growing Barki male lambs (4–5 months of age) were assigned randomly into four equal groups (6 lambs each). Four dietary treatments were the control group with 0% CM (CON) and three experimental groups where CM replaced 25% (CN_1_), 50% (CN_2_), and 75% (CN_3_) of cottonseed meal. There were no dietary effects (*P* > 0.05) on the lambs’ feed intake, average daily gain, and feed conversion ratio of the lambs. The dietary CM linearly decreased the concentrations of serum total proteins (*P* = 0.003), albumin (*P* = 0.010), globulin (*P* = 0.011), AST (*P* = 0.041), and urea (*P* = 0.001) in growing lambs. The levels of ALT and creatinine, however, were not significantly affected by dietary treatments (*P* > 0.05). Furthermore, serum triiodothyronine, thyroxine, and electrolyte concentrations were similar (*P* > 0.05) in different dietary groups. Dietary treatments significantly affected the values of ruminal pH and ammonia at 0 h (*P* = 0.003 and 0.048, respectively) and 3 h (*P* = 0.033 and *P* = 0.006, respectively) postfeeding. The CN_3_ group showed significantly higher concentrations of ruminal ammonia at 0 and 3 h postfeeding. Furthermore, dietary CM (CN_3_) significantly reduced the ruminal pH values at 0 and 3 h postfeeding. Meanwhile, dietary treatments did not affect the concentration of total VFAs in the ruminal fluid. In conclusion, CM can replace the cottonseed meal (up to 75%) in lamb diets without compromising their growth performance, thyroid function, and ruminal fermentation parameters.

## Introduction

Worldwide, the demand for high-quality protein has grown over time in order to meet human consumption. Among the conventional protein sources, animal protein is the most favored source of human consumption (Band et al. 2007). However, the increased meat consumption needs to be compensated by increased livestock production (Thornton 2010). In this context, the majority of individuals in low-income countries do not get enough protein and calories in their diet (Food and Agriculture Organization (FAO) 2012). Hence, considerable efforts have been made to improve livestock productivity in developing countries, which experience frequent fluctuations in the availability of natural feedstuffs and limited access to commercial diets. In order to fulfill the increased demand for dietary protein sources for livestock, several attempts have been made to explore less expensive and locally available alternatives.

Canola (*Brassica napus*) meal (CM), a byproduct of canola seed oil extraction, is a potential alternative dietary protein source. CM is abundant in protein and contains a lot of sulfur-containing amino acids (Newkirk et al. 2003). Furthermore, CM, which has a 40.9% crude protein, may provide farm animals with high-quality protein at a reasonable price (NASEM 2016). Compared to other oilseed meals, CM has been found to be a rich source of essential minerals such as Ca (0.9%), P (1.1%), Mg (0.6%), K (1.1%), Na (0.23%), and Cl (0.11%). The meal also contains about 8% ash (Paula et al. 2019). CM is thus a possible value-added product for canola residues (Inglis et al. 2021). Furthermore, CM is cheaper than other dietary protein sources, such as cottonseed meal and soybean meal (Maesoomi et al. 2006). Additionally, the antinutritive factors in cottonseed meal and the adverse environmental impacts of feeding high amounts of unavailable N are additional concerns that justify introducing a commercial alternative for cottonseed meal (Dolatkhah et al. 2020).

Unlike soybean meal, mechanically extracted CM may not be as effective as other sources of protein for ruminants due to its high rumen degradability (Broderick et al. 2016). This decreases the percentage of rumen bypass protein, as well as the rumen bacteria’s ability to utilize nitrogen. The high soluble protein concentration of CM contributes to its high rumen degradability (Newkirk 2015). In this context, approximately 30 µmol/g of glucosinolates has been found in CM (Newkirk et al. 2003). Due to their bitter taste, high concentration of glucosinolates is known to reduce dry matter intake (DMI) and consequently impair the thyroid function and the synthesis of thyroid hormone (Holst and Williamson 2004). In this context, some authors indicated that the inclusion of CM in the ruminant diets interferes with thyroid action due to myrosinase activity in CM (Lardy and Kerley 1994; Derycke et al. 1999). However, Rezaeipour et al. (2016) suggested that the use of canola meals in finishing lambs’ diets had no adverse effects on thyroid activity. Martineau et al. (2013) found that dairy cows fed CM had higher DMI and milk yield than many other frequently fed protein sources. According to a more recent study by Sekali et al. (2020), lamb diets could include CM without negatively affecting the animals’ growth performance, nutritional condition, or meat quality. A 35% greater response in terms of wool growth was observed in sheep-fed CM-supplemented diets (Coombe 1992).

Aspartate aminotransferase (AST) is an enzyme found in the heart and liver muscle and plays a vital role in the metabolism of amino acids (Stevanovic et al. 2015). Furthermore, the normal reference range of serum AST in sheep is 60–280 IU/L (Wang et al. 2015). Indeed, Paracova et al. (1998) reported that damage to cell membranes results in a high concentration of AST in the blood. Meanwhile, Sekali et al. (2020) indicated that none of the CM-containing diets had anti-nutritional compounds with cytotoxic properties. In ruminants, protein metabolism can also be monitored by the level of serum creatinine, which is positively correlated with muscle mass and negatively correlated with backfat thickness (Caldeira et al. 2007). We hypothesized that using CM in growing lamb diets will maintain the standard growth performance and normal metabolic functions. Therefore, the aim was to explain how replacing cottonseed meal with CM would affect growth performance, blood metabolites, thyroid function, and ruminal parameters of growing lambs.

## Materials and methods

The Nuclear Research Center, Atomic Energy Authority, Egypt, committee authorized the care and handling protocols for the experimental animals.

### Animals, management and experimental design

Twenty-four growing Barki male lambs, 4–5 months old, with a mean BW of 27.5 ± 1.5 kg, were assigned randomly into four equal groups (6 lambs each). The dietary groups were (1) control diet with 0% CM (CON), (2) diet with 5% CM (25% replacement of the cottonseed meal, CN_1_), (3) diet with 10% CM (50% replacement of the cottonseed meal, CN_2_), and (4) diet with 15% CN (75% replacement of the cottonseed meal, CN_3_). Before starting the experimental stage, animals received treatment for both internal and exterior parasites (Ivermectin 1% and albendazole). According to NRC (2007), the experimental diets were isonitrogenous and adjusted to meet the lambs finishing dietary requirements (age 4–7 months). The forage-to-concentrate ratio was 30:70 (DM basis), and the total mixed ration was provided twice daily at 7:00 and 14:00. Lambs were kept in separate standard pens (3.4 m^2^) with bedded straw. Also, lambs had free access to clean drinking water at all the times. The lambs were then adapted to the experimental diets and pens for 10 days before measurements commenced. The chemical composition of CM, cotton seed meal, and the formulated experimental diets is illustrated in Table 1 (AOAC 1997). Canola seeds were cultivated, harvested, and then mechanically pressed to obtain the canola meal.

**Table 1:** Ingredients and approximate chemical composition of the experimental diets fed to growing lambs

Item	Diets					
	^1^CON	^2^CN_1_	^3^CN_2_	^4^CN_3_	^5^CN	^6^CSM
Ingredients (%)						
Yellow corn	54.0	54.0	54.0	54.0		
Cotton seed meal	20.0	15.0	10.0	5.0		
Canola meal	0.0	5.0	10.0	15.0		
Soybean meal	10.0	10.0	10.0	10.0		
Wheat bran	13.0	13.0	13.0	13.0		
NaCl	1.0	1.0	1.0	1.0		
Dicalcium phosphate	1.5	1.5	1.5	1.5		
Mineral mixture	0.4	0.4	0.4	0.4		
Vit. AD_3_E	0.1	0.1	0.1	0.1		
Chemical composition (DM basis, %)						
Dry matter (DM)	87.66	87.65	87.63	87,62	92.77	93.00
OM	93.63	93.58	93.53	93.48	93.12	94.13
CP	16.89	16.89	16.89	16.89	29.05	29.02
CF	7.26	6.76	6.26	5.76	12.98	22.96
EE	4.73	4.82	4.91	5.01	13.80	11.93
NFE	64.76	65.12	65.47	65.82	37.29	30.22
Ash	6.37	6.42	6.47	6.52	6.88	5.87

^1^control group (0 canola meal); ^2^25% canola meal; ^3^ 50% canola meal; ^4^75% canola mea; ^5^canola meal; ^6^cotton seed meal. *OM* organic matter, *CP* crude protein, *CF* crude fiber, *EE* ether extract, *NFE* nitrogen-free extract

Mineral mixture: 30 mg Zn as Zn SO4.7H2O; 20 mg Mn as MnSO4.H2O; 0.5 mg I as KI; 0.1 mg, Co as CoCl2; 0.1 mg, Se as Na2SeO3,1500 IU vitamin A; 250 IU vitamin D, and 16 IU vitamin E per Kg DM

### Feed intake and growth performance

A 98-day feeding study was conducted. Prior to feeding, the offered feed was weighed, and refusals were gathered. The feed intake (FI) was then calculated using the difference between the feed offered and the refusals. Each lamb was weighed before morning feeding every 2 weeks over the experimental period, and the average daily gain (ADG) was determined as follows:

$$ADG=W\left(T\right)-W\left({t}_{0}\right)/ T-{t}_{0}$$

where *W*(*T*) is the final *BW*, and *W*(*t*_0_) is the initial BW, and *t*_0_ is the starting time. *T* is the final time (98th day). The feed conversion ratio (FCR) for the entire feeding period was then calculated using the ratio of FI to weight gain.

### Blood sampling and serum biochemical parameters and thyroid hormones

On a monthly basis, before morning feeding, 3-mL blood samples were collected from the jugular vein by venipuncture using an 18-gauge needle into vacutainer tubes (Red top Vacuette® Serum Clot Activator tubes). The samples (five lambs in each group) were centrifuged at 3000 rpm for 15 min before the serum was transferred into a polypropylene tube and kept at − 20 C°. Using commercial kits, the following indices were measured: alanine aminotransferase (ALT), urea, creatinine, aspartate aminotransferase (AST), total proteins, and albumin (Spinreact, Esteve De Bas, Girona, Spain). The difference between total protein and the corresponding value of albumin was used to compute the globin level. Triiodothyronine (T_3_) and thyroxine (T_4_) serum concentrations were measured following the instructions provided with the RIA-coated tubes (RIAKEYTUBE II®, Republic of Korea).

### Blood electrolytes

On the last day of the study, 2-mL blood samples were collected from the jugular vein by venipuncture using an 18-gauge needle into vacutainer tubes (Red top Vacuette® Serum Clot Activator tubes), and serum was separated (3000 rpm for 15 min). Colorimetrically, the concentrations of serum Ca (o-cresolphthalein in alkaline medium), Na (O-nitrophenyl-β-D-glucoside), Mg (xylidyl blue), Ph (phosphomolybdate), and K (pyruvate kinase, POTASSIUM-LQ) were evaluated (Spinreact, Esteve De Bas, Girona, Spain).

### Ruminal parameters (ruminal fermentation parameters)

At the end of the feeding trial, rumen liquor samples were obtained from three animals in each group. The samples were taken from lambs by stomach tube with a vacuum pump at 0, 3, and 6 h after the morning feeding, and the samples were controlled to avoid contamination with saliva. After collecting rumen liquor, the pH values were immediately measured using a digital pH meter (pH Pen, 8686 AZ, Taichung, Taiwan). The rumen fluid was collected, filtered through four layers of cheesecloth, and equipped to measure the following parameters: ammonia (50 ml of rumen fluid was acidified with one ml of (vol/vol) H_2_SO4 50%) and VFAs (5 ml of rumen fluid was mixed with one ml of 250 g/L meta-phosphoric acid), all while being kept at − 20 °C pending further analysis. According to the protocol of Broderick and Kang (1980), the concentration of ruminal ammonia-N was measured using a colorimetric phenol-hypochlorite technique. The colorimeter was equipped with 630 nm filters and a 4 mm flowcell. The results of the phenol-hypochlorite assay were recorded on chart paper calibrated in percent transmittance (%T), and absorbance was calculated from %T. The standard program (Texas Instruments, Richardson, TX) was applied to convert %T to absorbance units and calculated slopes of standard curves. The concentration of total VFAs was determined by the steam distillation method using the Markham micro-distillation apparatus (Warner 1964).

### Statistical analyses

The IBM SPSS software’s GLM procedures were used to analyze the collected data (Version 16.0; IBM Corp., NY, USA). Barki lambs (6 lambs per treatment) were assigned randomly into four equal groups (CON, CN_1_, CN_2_, and CN_3_). Furthermore, the experimental unit of this design was the individual animal. The polynomial contrasts were included to evaluate linear and quadratic responses to the CM level. Using the following statistical model, data on growth performance, blood electrolytes, and ruminal parameters were analyzed:

$${Y}_{\mathrm{ij}}=\mu +{T}_{\mathrm{i}}+{e}_{\mathrm{ij}}$$

where:

*Y*_ij_: the dependent variable.
*μ*: the population mean.
*T*_i_: the effect of CM level (i = CON, CN_1_, CN_2_, and CN_3_).
*e*_ij_: random error.

Data on blood biochemical parameters and thyroid hormones were evaluated in a repeated measurements model. The model considers the effects of time, diet, and their interactions. To compare the means, Duncan’s multiple range test was applied. The cutoff for significance was *P* < 0.05.

## Results

### Feed intake and growth performance

Table 2 shows the effects of replacing cottonseed meal with CM on FI and growth performance in Barki lambs. The lambs’ feed consumption, ADG, and FCR did not change due to the dietary treatment (*P* > 0.05). The lambs’ FCR varied from 6.11 to 6.98 (*P* = 0.640).

**Table 2:** Effect of replacing cottonseed meal with canola meal on growth performance of growing lambs

Item	*Experimental groups				^5^SEM	*P* value	
	^1^CON	^2^CN_1_	^3^CN_2_	^4^CN_3_			
						Linear	Quadratic
Initial BW, kg	27.0	27.0	27.8	27.2	0.39	0.749	0.634
Final BW, kg	48.7	49.9	47.5	47.5	0.73	0.394	0.701
^6^ADFI, kg	1.35	1.37	1.32	1.36	0.06	0.916	0.842
^7^ADG, kg	0.221	0.232	0.201	0.207	0.02	0.161	0.860
^8^FCR	6.11	6.70	6.98	6.78	0.49	0.640	0.712

^*^^1^control group (0 canola meal); ^2^25% canola meal; ^3^ 50% canola meal ^4^75% canola meal; ^5^standard error of mean; ^6^average daily feed intake; ^7^average daily gain; ^8^feed conversion ratio

^*^Sample size was 6 per each group

### Blood metabolites, thyroid hormones, and electrolytes

Table 3 shows how the partial replacement of cottonseed meal with CM affected the blood metabolites of growing lambs. The dietary CM linearly decreased the levels of serum total proteins (*P* = 0.003), albumin (*P* = 0.010), globulin (*P* = 0.011), AST (*P* = 0.041), and urea (*P* = 0.001) in growing lambs. However, the effects of dietary treatments on ALT and creatinine levels were insignificant (*P* > 0.05). The levels of triiodothyronine and thyroxine in growing lambs were unaffected by the dietary treatment, as indicated in Table 4 (*P* > 0.05). Furthermore, the concentrations of serum electrolytes (Ca, P, K, Mg) were similar (*P* > 0.05) in different groups (Table 5).

**Table 3:** Effect of replacing cottonseed meal with canola meal on blood biochemical parameters of growing lambs

Item	*Experimental groups				^5^SEM	*P* value		
	^1^CON	^2^CN1	^3^CN2	^4^CN3		^6^CN		^7^ T
						Linear	Quadratic	
Total protein, g/dl	8.01^a^	8.0^a^	7.19^b^	7.41^b^	0.169	0.003	0.499	0.001
Albumin, g/dl	3.93^a^	3.94^a^	3.61^b^	3.67^b^	0.082	0.010	0.082	0.031
Globulin, g/dl	4.12^a^	4.06^a^	3.57^b^	3.73^ab^	0.128	0.011	0.411	0.001
^8^A/G ratio	0.98	1.01	1.0303	0.99	0.036	0.565	0.395	0.089
^9^ALT, U/L	16.21	14.83	16.12	13.99	2.21	0.598	0.869	0.111
^10^AST, U/L	104.9^ab^	116.6^a^	91.9^b^	96.8^b^	5.01	0.041	0.546	0.003
Creatinine, mg/dl	0.95	0.94	0.82	0.87	0.041	0.061	0.616	0.001
Urea, mg/dl	65.58^a^	66.59^a^	51.15^b^	40.24^c^	3.32	0.001	0.092	0.005

^*^^1^control group (0 canola meal); ^2^25% canola meal; ^3^ 50% canola meal ^4^75% canola meal; ^5^standard error of mean; ^6^effect of canola meal supplement; ^7^effect of time; ^8^albumin/globulin ratio; ^9^alanine aminotransferase; ^10^aspartate aminotransferase. ^a,b,c^Means with different superscripts in each row are significantly differed

^*^Sample size was 5 per each group

**Table 4:** Effect of replacing cottonseed meal with canola meal on the levels of thyroid hormones in growing lambs

Item	*Experimental groups				^5^SEM	*P* value		
	^1^CON	^2^CN1	^3^CN2	^4^CN3		^6^CN		^7^ T
						Linear	Quadratic	
^8^T_3_, mg/dl	1.41	1.36	1.79	1.32	0.108	0.781	0.171	0.253
^9^T_4_, mg/dl	97.6	97.4	110.7	107.6	6.62	0.162	0.830	0.001

^*^^1^control group (0 canola meal); ^2^25% canola meal; ^3^ 50% canola meal ^4^75% canola meal; ^5^standard error of mean; ^6^effect of canola meal supplement; ^7^effect of time; ^8^triiodothyronine; ^9^thyroxine

^*^Sample size was 5 per each group

**Table 5:** Effect of replacing cottonseed meal with canola meal on blood electrolytes of growing lambs

Item	*Experimental groups				^5^SEM	*P* value	
	^1^CON	^2^CN_1_	^3^CN_2_	^4^CN_3_			
						Linear	Quadratic
Ca, mmol/L	9.35	8.50	8.48	8.93	0.18	0.480	0.109
Ph. mmol/L	4.86	5.45	5.80	5.95	0.21	0.272	0.757
Mg, mg/dl	2.08	1.83	1.87	1.95	0.07	0.666	0.363
K, mmol/L	4.17	5.14	5.27	5.13	0.23	0.174	0.249
Na, mmol/L	146	147	146	145	3.8	0.789	0.627

^*^^1^control group (0 canola meal); ^2^25% canola meal; ^3^ 50% canola meal ^4^75% canola meal; ^5^standard error of mean

^*^Sample size was 5 per each group

The effects of time on the levels of serum total protein, albumin, globulin, AST, creatinine, urea, and thyroxin were significant (*P*˂0.05). Meanwhile, the effects of time on serum ALT and triiodothyronine levels were non-significant (*P* ˃ 0.05). In this context, serum levels of total protein, globulin, urea, and thyroxin showed significant effects for the interaction between dietary treatment and time (*P*˂0.05).

### Ruminal parameters (ruminal fermentation parameters)

Table 6 shows the impact of the partial replacement of cottonseed meal with CM on ruminal parameters in growing lambs. Dietary treatments significantly affected the ruminal pH and ammonia values at 0 h (*P* = 0.003 and 0.048, respectively) and 3 h (*P* = 0.033 and 0.006, respectively) postfeeding. The CN_3_ group showed significantly higher concentrations of ruminal ammonia at 0 and 3 h postfeeding compared to other experimental groups. Furthermore, dietary CM (CN_3_) linearly reduced the ruminal pH values at 0 and 3 h postfeeding compared to the CON group. Meanwhile, dietary treatments did not impact on the levels of total VFAs in ruminal fluid.

**Table 6:** Effect of replacing cottonseed meal with canola meal on ruminal fermentation parameters of growing lambs

Parameter	Time (hour)	*Experimental groups				^5^SEM	*P* value	
		^1^CON	^2^CN_1_	^3^CN_2_	^4^CN_3_			
							Linear	Quadratic
Ammonia (Mg/100 ml)	0	33.6^b^	42.0^ab^	36.4^ab^	45.7^a^	2.80	0.048	0.878
	3	46.7^b^	57.9^b^	50.4^b^	71.9^a^	3.39	0.006	0.248
	6	47.6	64.4	42.9	53.2	3.14	0.831	0.510
^6^TVA (Meq/100 ml)	0	4.50	4.10	4.10	4.33	0.11	0.217	0.005
	3	7.0	7.90	8.20	7.90	0.41	0.491	0.537
	6	8.23	9.60	8.23	8.40	0.38	0.556	0.094
pH	0	7.98^a^	7.78^ab^	7.49^bc^	7.30^c^	0.08	0.003	0.688
	3	7.27^a^	7.01^ab^	6.86^ab^	6.93^b^	0.11	0.033	0.973
	6	6.76	6.22	6.42	5.29	0.09	0.101	0.581

^*^^1^control group (0 canola meal); ^2^25% canola meal; ^3^ 50% canola meal ^4^75% canola meal; ^5^standard error of mean; ^6^total volatile fatty acids. ^a,b,c^Means with different superscripts in each row are significantly differed

^*^Sample size was 3 per each group

## Discussion

The goal of today’s worldwide challenge is to maximize the utilization of protein sources in different production systems, with developed countries primarily focusing on the quality rather than the quantity of meat produced (Gulati et al. 2005). In the present study, the similar feed intake could be attributed to the isonitrogenous nature of the experimental diets. In this context, Dabiri (2016) suggested that dietary energy level may be one of the most important factors regulating the FI when growing ruminants fed adequate protein sources. This may be one of the possible causes of the lack of variations in FCR and ADG between experimental groups. Consistent with these findings, no dietary effects were seen (*P* > 0.05) on the FI, ADG, and FCR (5.42–5.89) in Meatmaster lambs when CM partially (50%) or completely replacing soybean meal (Sekali et al. 2020). This range of FCR is quite better than that reported in the current study, probably due to breed differences and the inclusion of heat-treated CM in lamb diets. Additionally, Maesoomi et al. (2006) found no variations in dry matter intake between dietary regimens when CM partially (50%) or wholly replaced cottonseed meal in the feed of Holstein cows. Others have observed similar results using sunflower meal partially (50%) or completely replacing the soybean meal in Awassi lamb diets (Irshaid et al. 2003). Contrary to these findings, others noticed that FI and feed efficiency of fattening lambs were significantly improved when CM was included in the diets at 12% (Wiese et al. 2003) and 28% (Khalid et al. 2011). They suggested that the canola meal-based diet promotes the anabolic processes in crossbred fattening lambs.

Although the isonitrogenous diets were consumed in similar amounts by each treatment group, the dietary CM linearly reduced the levels of blood total proteins, albumin, and globulin, but the growth performance of growing lambs was unaffected by this. This reduction in the levels of blood metabolites may be attributed to the high rumen degradability of mechanically extracted CM as an alternative protein source for ruminant animals (Broderick et al. 2016). In this context, plasma concentrations of some non-essential amino acids can decrease due to their utilization for hepatic gluconeogenesis (Miettinen and Huhtanen 1997). The high soluble protein content of CM contributes to its high rumen degradability (Newkirk 2015). Moreover, protection of dietary proteins led to lower rumen degradability and higher concentrations of proteins escaping to the abomasum and the small intestine and consequently higher absorption of dietary amino acids, which lead to high level of plasma protein (Abdel-Ghani et al. 2011). Sekali et al. (2020), on the other hand, hypothesized that dietary CM had no impact on the levels of total protein and albumin in growing lambs. Additionally, they anticipated that lambs fed a heat-treated CM would have greater serum protein level as a sign of higher quantities of absorbed essential amino acids from the post-rumen, primarily facilitated by higher bypass protein levels. The heart muscles and liver contain the enzyme aspartate aminotransferase, which is crucial for the normal metabolic activity of amino acids (Stevanovic et al. 2015). In fact, Paracova et al. (1998) revealed that a high plasma AST level is caused by damage to cell membranes. Compared to the standard reference range of sheep (60–280 IU/L), the serum AST activities in the current study are acceptable (Wang et al. 2015). Also, the dietary CM linearly decreased the concentrations of serum AST in growing lambs. Therefore, our results showed that none of the dietary regimens using CM had anti-nutritional chemicals having cytotoxic effects.

In the present study, dietary CM linearly decreased serum urea concentration in growing lambs. Similar to this, Mustafa et al. (1997) assessed the differences between feeding cows CM and soybean meal and found that feeding cows CM resulted in a 13% drop in blood urea N. Martineau et al. (2014) also indicated that feeding CM lowered the concentrations of blood urea N in dairy cattle, suggesting an improved whole-body N utilization efficiency by the ruminant animals and that more dietary protein was utilized (Broderick et al. 2008). In this context, around 10 to 40% of N consumed in the feed is recycled back to the digestive tract as urea from saliva where it can be used again for microbial synthesis (Bach et al. 2005).

Growing lambs on diets containing CM had stable metabolic conditions and a moderate level of homeostasis, as evidenced by the levels of serum electrolytes, triiodothyronine, and thyroxine not different among dietary groups. Rezaeipour et al. (2016) found that varying levels of dietary CM had no significant effects on thyroid hormone secretion (triiodothyronine and thyroxin) in Atabay finishing lambs, which accords with our findings. Maesoomi et al. (2006) also reported similar levels of plasma thyronines in mid-lactation dairy cows fed diets containing CM instead of cottonseed meal. In this context, it has been reported that CM contains about 2 mol/g glucosinolates (Newkirk et al. 2003). Furthermore, excessive glucosinolate concentrations are known to impair thyroid function by preventing the synthesis of thyroid hormones (Holst and Williamson 2004). With an increase in rapeseed meal in the diets, a linear drop in thyroxin level was reported in Holstein calves (Lardy and kerley 1994). However, Martineau et al. (2013) showed increased FI and stable thyroid function in dairy cows fed CM compared to other frequently fed protein sources.

Compared to other experimental groups in the present research, dietary CM (CN_3_) significantly elevated the levels of ruminal ammonia at 0 and 3 h postfeeding. Because CM has a balanced amino acid profile and a high degree of crude protein degradability, Piepenbrink and Schingoethe (1998) hypothesized that rumen degradable protein (RDP) from CM has promoted microbial protein synthesis. Similarly, Krizsan et al. (2017) suggested that the leading cause of the increased ruminal ammonia in CM-supplemented dairy cows is likely due to increased proteolysis of a greater amount of available feed protein and hence increased deamination of amino acids to NH3. Meanwhile, Brito et al. (2007) found no variations in ruminal ammonia concentrations between dairy cow diets supplemented with CM, cottonseed meal, or soybean meal. Using an in vitro dual flow continuous culture system, Paula et al. (2017) did not note any variations in ruminal ammonia concentration while feeding diets containing CM compared to soybean meal. Compared to soybean meal, cows fed CM diets had reduced ammonia levels (Broderick et al. 2015). Also, different dietary treatments in the current trial did not affect the concentration of total VFAs in the ruminal fluid. Similarly, Christen et al. (2010) found no variation in any VFA traits among different protein supplements, soybean meal, CM, or dried distillers grains (DDGS) in isonitrogenous diets. Regarding total VFAs, Broderick et al. (2015) failed to distinguish any differences between CM and soybean meal diets. Furthermore, no variations in any VFA traits were seen in several trials comparing ruminal fermentation parameters in dairy cows fed diet supplements with CM vs. soybean meal (Li et al. 2013; Paula et al. 2018; Brandao et al. 2018). Comparing CM vs. DDGS, Mulrooney et al. (2009) found no variation in total or individual VFA concentrations.

Compared to the CON group in this study, dietary CM (CN_3_) decreased the ruminal pH values at 0 and 3 h post feeding. Krizsan et al. (2017) noticed that ruminal pH is linearly decreased with increased dietary CM level in lactating Nordic red cows, which is consistent with our findings. Meanwhile, Tajaddini et al. (2021) suggested that different levels of dietary CM did not change the ruminal pH values at 0 and 3 h postfeeding in lactating goats. In lactating cows, Broderick et al. (2015) also reported no differences between CM and soybean meal diets in terms of ruminal pH values.

## Conclusion

It could be concluded that CM can replace the cottonseed meal (up to 75%) in lamb diets without compromising their growth performance, thyroid function, and ruminal fermentation parameters. Furthermore, dietary CM increased the concentrations of ruminal ammonia, which indicated higher degradation of CM protein in growing lambs.

## Data Availability

All data generated or analyzed during this study are included in this published paper.
